# Enhanced Flame Retardancy of Rigid Polyurethane Foams by Polyacrylamide/MXene Hydrogel Nanocomposite Coating

**DOI:** 10.3390/ijms232012632

**Published:** 2022-10-20

**Authors:** Bin Chen, Lizhong Yang

**Affiliations:** State Key Laboratory of Fire Science, University of Science and Technology of China, Jinzhai Road 96, Hefei 230026, China

**Keywords:** rigid polyurethane foams, flame-retardant coating, poly acrylamide hydrogel, MXene, nanocomposites

## Abstract

Rigid polyurethane foam (RPUF) has been widely used in many fields, but its high flammability and frequent release of large amounts of toxic smoke during combustion limit its application. Hydrogel coatings, as a kind of environmentally friendly material, contain large amounts of water, which is beneficial to flame retardance of RPUF. MXene, as a two-dimensional inorganic nanomaterial, possesses a large specific surface area and good thermal stability, performing well in smoke suppression and as a physical barrier for flammable gas products and heat. Herein, to address the fire hazards of RPUF, MXene nanosheets were first grafted with double bonds, and then introduced into a polyacrylamide hydrogel system by radical polymerization to prepare MXene-based hydrogel coating (PAAm-MXene). The flame-retardant RPUF (coated RPUF) was prepared by painting the PAAm-MXene coating onto RPUF surface. The dispersion of modified MXene nanosheets (m-MXene) in hydrogels is improved compared with pristine MXene, and the addition of m-MXene contributes to the thermal stability enhancement of PAAm-MXene. Cone calorimetry, water retention test, and open flame combustion test were used to study the flame retardancy, smoke suppression, and water retention of flame-retardant RPUF. The coated RPUF exhibited significant flame retardancy, including reduced peak heat release rate (pHRR) at a maximum by 25.8%, and total heat release rate (THR) at a maximum by 24.6%, and total smoke production at a maximum by 38.9%. The results show that both MXene and m-MXene can improve the flame retardancy, smoke suppression, and water retention of hydrogels, but m-MXene has a better smoke suppression effect than MXene. That can be ascribed to the better dispersion of m-MXene than pristine MXene. The detailed performance improvement mechanisms are proposed. This work will not only improve the flame retardancy of RPUF, but also promotes the exploration of new flame-retardant strategies for RPUF.

## 1. Introduction

Rigid polyurethane foam (RPUF), as an excellent thermal insulation polymeric material, exhibits very low thermal conductivity and good mechanical performance but is extremely flammable and releases a lot of heat and toxic fumes during the combustion process, which tends to cause serious casualties and property loss [1]. To broaden its application, flame retardance and smoke suppression treatments of RPUF are very important.

As is well known, the flame retardance strategy of RPUF includes the “overall type” and “coating type”. The “overall type” strategy is mainly achieved by introducing additive flame retardants and reactive flame retardants to polymers [2]. Although this strategy can improve the flame retardancy of RPUF to some extent, the additive flame retardants tend to move from inside to outside of polymers in use, leading to the loss of flame retardancy; the preparation process of reactive flame retardant is complex and expensive. Furthermore, to obtain a good flame retardancy, high amounts of additive flame retardants or reactive flame retardants are required, which often deteriorate the mechanical properties and thermal insulation performance of RPUF. Compared with the “overall type” strategy, the “coating type”, as a post-processing method, can not only improve flame retardancy of RPUF, but also maintains the excellent inherent properties of the RPUF substrate including thermal insulation property and flame retardancy, because it does not affect the structure and components of the RPUF matrix. Moreover, the surface properties of the coating are easy to adjust [3,4,5].

In the case of flame-retardant coatings, the intumescent coating is very helpful which enhances the flame retardancy of RPUF by forming intumescent char during combustion [6,7]. The char inhibits the transfer of flammable gas products, oxygen, and heat, protecting the underlying substrate. However, the intumescent flame-retardant coating tends to generate fly ash or smoke during combustion. To address that, the nanocomposite coating is a good choice which can enhance flame retardancy, and meanwhile reduces the toxic fumes released with small amounts of nanoadditives through the physical barrier effect and the catalytic effect of inorganic nanosheets. In terms of flame retardancy for the nanocomposite coating, the nanoadditive plays a very important role [8,9,10].

MXene is a new type of two-dimensional nanomaterial with large surface areas, good thermal stability, smoke suppression, mechanical properties, etc., which makes MXene an ideal flame-retardant nanoadditive for polymer nanocomposites [11,12,13,14]. At the same time, the surface of MXene has a variety of functional groups attached, which enables MXene potential to be organically modified on the surface. The introduction of organically modified MXene into the flame-retardant coating system will theoretically affect the flame retardant and smoke suppression performance and mechanical properties of the flame-retardant coating [15,16,17,18,19]. Nonetheless, according to the available references, the addition of only nanoadditives for nanocomposite coating cannot achieve self-extinguishment properties [20,21,22,23]. Hydrogel coatings, as green materials, contain high contents of water, and have excellent properties such as high viscosity, strong hydrophilicity, and self-healing. When exposed to flames or high temperatures, the vaporization of water from the hydrogel would benefit the fireproof properties of the substrate [4,21,22].

In this work, in order to achieve a self-extinguished RPUF with good smoke suppression, MXene was firstly grafted with double bonds, obtaining modified MXene (m-MXene). The m-MXene was then added to the polyacrylamide hydrogel through radical polymerization. The as-fabricated polymer nanocomposite hydrogel coating was painted onto the surface of RPUF. The organic modification was conductive for the dispersion of MXene nanosheets in the hydrogel system. After property studies, the coated RPUF not only showed significantly enhanced flame retardancy and smoke suppression, but also retained the excellent mechanical properties and thermal insulation properties of RPUF. The detailed mechanism is proposed.

## 2. Results and Discussion

### 2.1. Characterization of m-MXene

**Figure 1:**
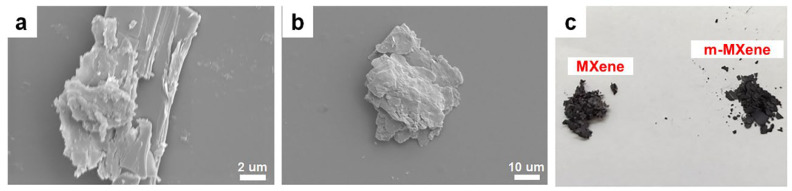
(**a**) SEM image of MXene; (**b**) SEM image of m-MXene; (**c**) Optical image of MXene and m-MXene.

After ultrasonic treatment of MXene to obtain a few layer MXene flakes of Ti_3_C_2_Tx, the MXene (Ti_3_C_2_Tx) was then grafted with C=C bonds by the modifier, isocyanoethyl methacrylate, to perform surface functionalization. Figure 1a,b shows the SEM images of MXene and m-MXene, respectively. From Figure 1a, the MXene nanosheets have an accordion-like feature structure separated from each other, which indicates that Ti_3_AlC_3_ has been successfully etched, and the Al layer is etched. It can be seen from Figure 1b that the lamellar structure of MXene remains after functional modification, but the boundary becomes blurred due to the bonding force between lamellae weakening after surface functional modification expansion, so that the MXene sheet stacking structure is destroyed. It can be seen from the optical photos that MXene and m-MXene have a slight difference in color (as shown in Figure 1c); MXene is gray in color, while the modified MXene is black in color.

**Figure 2:**
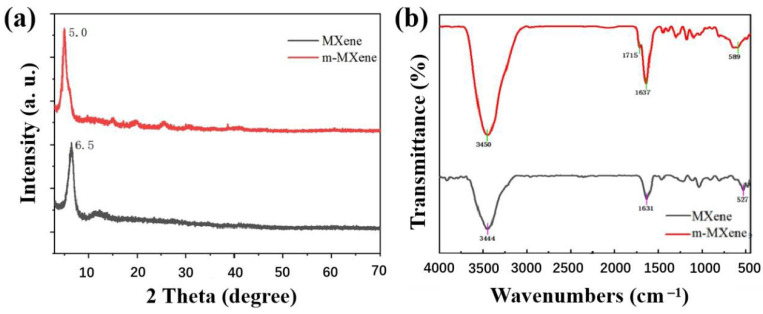
(**a**) XRD patterns of MXene and m-MXene; (**b**) FTIR spectra of MXene and m-MXene.

From the XRD pattern (Figure 2a), compared with MXene, m-MXene has the highest diffraction peak shifted to a lower angle, from 6.5° to 5.0°. Similar characteristic peaks can prove that the structure of the TiC_2_Tx layer is not destroyed after being treated by the modifier, but the interlayer spacing of m-MXene is larger than that of MXene due to the introduction of some modified molecules. This also indirectly proves that MXene is successfully modified. The chemical structure of m-MXene was analyzed by FTIR spectroscopy (as shown in Figure 2b). The most prominent peaks of MXene are the -OH vibration peak (the absorption peak of -OH is 3700~3200 cm^−1^) and the vibration peak of the Ti–O bond at 527 cm^−1^. For the m-MXene, there are new vibration peaks at 1715 cm^−1^ and 1637 cm^−1^; these two vibration peaks correspond to the carbonyl C=O bond vibration peak (carbonyl generally appears at 1870~1600 cm^−1^ region) and the vibration peaks of C=C bonds (C=C bonds generally appear in the 1650~1600 cm^−1^ region), respectively. In addition, the intensity of the -OH vibrational peak was significantly increased, and there was no significant vibrational peak of the Ti–O bond. According to the above analysis, the surface organic modification of MXene is successful.

### 2.2. Morphology and Structure of Coated RPUF

**Figure 3:**
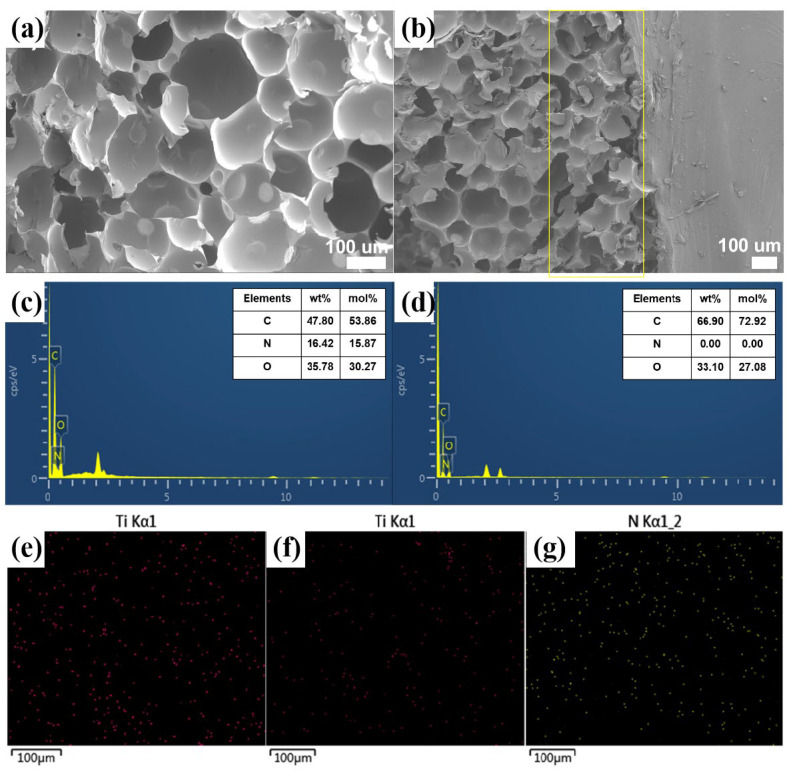
(**a**) SEM image of RPUF surface; (**b**) SEM image of PAAm-MXene Coated RPUF surface; (**c**) EDX N element distribution map on RPUF surface; (**d**) EDX Ti element distribution map on PAAm Coated RPUF surface; (**e**) Distribution of EDX Ti elements on the surface of PAAm-MXene Coated RPUF; (**f**) Distribution of EDX Ti elements on the surface of PAAm-m2 Coated RPUF; (**g**) Distribution of EDX N elements on the surface of PAAm-m2 Coated RPUF.

In order to evaluate the surface coating properties of Coated RPUF, the SEM and EDX were used to study the surface morphology and element distribution of the coatings. Figure 3a shows the morphology of pure RPUF under SEM. RPUF has a regular closed-cell structure, which makes RPUF have excellent thermal insulation performance, and at the same time makes it difficult for the precursor liquid of the coating to penetrate the interior of RPUF, resulting in changes in the performance of RPUF itself. Figure 3b shows the interface of PAAm-MXene Coated RPUF. The flame-retardant coating and RPUF are closely connected. This may be because the porous structure of the foam surface allows a part of the hydrogel precursor liquid to penetrate the surface, resulting in the surface of the hydrogel coating and RPUF becoming tightly bound. The internal structure of RPUF has not changed, so the application of the flame-retardant coating has little effect on the performance of RPUF itself. Meanwhile, it can be seen from the EDX images (Figure 3d–f) that the Ti element appears on the surface of PAAm-MXene Coated RPUF and PAAm-m2, which indicates that the coating containing MXene was successfully coated on the surface of RPUF. From the comparison of Figure 3e,f, the functional modification of MXene reduces the agglomeration of MXene molecules, which improves the dispersion of MXene molecules in polyacrylamide hydrogel coatings. Moreover, it can be seen from Figure 3g that the N element on the surface of PAAm-m2 exists and is uniformly distributed, this is because the functionalized modified MXene contains a nitrogen element, which further verifies the successful modification of MXene and the modification of MXene. It helps to improve the dispersibility of MXene in the hydrogel system.

### 2.3. Thermal Property of Coated RPUF

**Figure 4:**
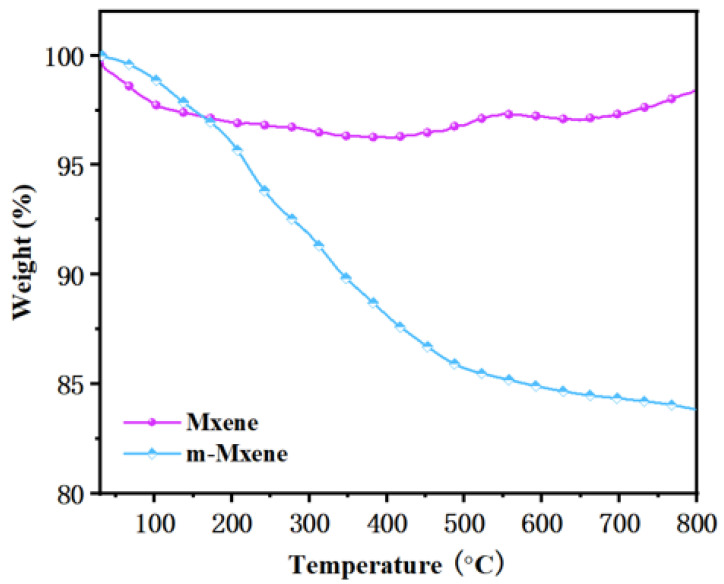
TGA plots of MXene and m-MXene under nitrogen.

Figure 4 shows the thermal degradation curves of MXene and m-MXene after lyophilization for 24 h to remove moisture in the sheet. The conditions of this experiment were to increase the temperature from 20 °C to 800 °C at a heating rate of 20 °C min^−1^ in a nitrogen atmosphere. The mass loss of MXene at the end of the test is less (1.61%), and its mass loss is mainly due to the degradation of a large number of end groups on the surface of MXene; the mass loss of m-MXene is larger (16.16%), and its mass loss may be the degradation of grafted groups after the MXene surface modification. This characteristic of m-MXene may have a “negative effect” on the flame retardancy of the polyacrylamide hydrogel flame-retardant coatings.

**Figure 5:**
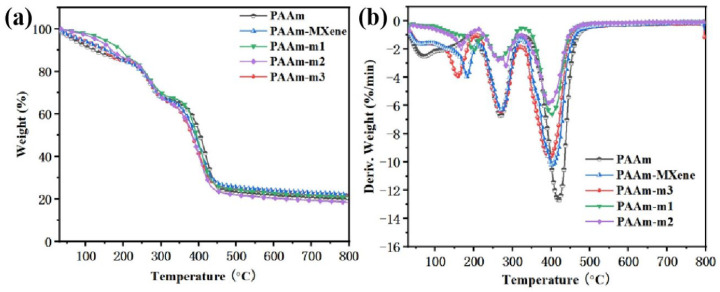
(**a**) TGA and (**b**) DTG curves of coatings under nitrogen atmosphere.

The thermal decomposition of PAAm, PAAm-MXene, PAAm-m1, PAAm-m2, and PAAm-m3 was carried out from 20 °C to 800 °C in a nitrogen atmosphere, and the heating rate was 20 °C min^−1^. The corresponding curve can be observed in TGA and DTG (Figure 5).

From Figure 5a, the TGA curves of these five coatings roughly overlap, and the weight loss process can be roughly divided into three processes from the changing trends and inflection points of the TGA curves. The temperature range of the first stage is 20–300 °C. Since the samples have been freeze-dried for 48 h before the test, the weight loss of the first stage may be caused by the decomposition of small molecular substances in the samples. In this stage, the drop rate of the rest of the nanocomposite hydrogel coatings is slowed compared with that of PAAm, which indicates that the addition of MXene can improve the thermal stability of the coatings at a low temperature. Comparing PAAm-MXene and PAAm-m2, when the addition amount of MXene is 10 mg, the surface functional modification of MXene can improve the thermal stability of the coating. It can be seen from the comparison of PAAm-m1, PAAm-m2, and PAAm-m3 that with the increase in the addition amount, the thermal stability of the coating becomes worse, which is caused by the “negative effect” of MXene on the coating. The temperature range in the second stage is 300–400 °C. The quality reduction in the coating at this stage is mainly caused by the thermal decomposition of the side chain of acrylamide. It can be seen from the TG diagram that the decrease rate of PAAm at this stage is slower than the other nanocomposite hydrogels. From the comparison of PAAm-m1, PAAm-m2, and PAAm-m3, with the increase in the addition amount of MXene, the rate is becoming faster and faster. The functional modification of MXene at this stage has little effect on the thermal stability of the coating, which can be drawn from the comparison of PAAm-MXene and PAAm-m2. Therefore, the reason for the phenomenon may be due to the poor high temperature stability of MXene. The third stage is 400–800 °C, which is the skeleton of the coating and the further decomposition of the imine structure and the breaking of the C=C bond until the coating is completely burned into char.

From the image of DTG (Figure 5b), there are two peaks. Combined with the analysis of TGA, the first peak is mainly due to the decomposition of small molecular substances and the condensation of water. The network of the glue coat begins to break down and a char layer forms on the surface of the sample. During the period from the first peak to the second peak, the side chain of acrylamide begins to decompose and the hydrogel begins to burn. The second peak occurs because the side chain of acrylamide is completely decomposed and the backbone begins to break. Compared with the PAAm coating, the maximum weight loss rate of the nanocomposite hydrogel coating is significantly reduced, since the MXene migrates onto the contact surface with the heat source due to the low energy of the surface during the carbonization process. The formed char layer can better isolate the mixing of oxygen and the transfer of heat [1,24,25,26]. Compared with the hydrogel coating added with m-MXene, PAAm-MXene has a higher maximum weight loss rate, which may be due to the better dispersibility of m-MXene in the polyacrylamide hydrogel system, which leads to char formation. The distribution of MXene in the char layer is more uniform and the thermal stability is better. The comparison of the maximum weight loss rate of PAAm-m1, PAAm-m2, and PAAm-m3 shows that the maximum weight loss rate of PAAm-m3 is significantly larger than the other two, which may be due to the “negative effect” brought about by the addition of MXene [27]. Due to the excellent thermal conductivity of MXene, the excessive addition of MXene increases the thermal conductivity of the hydrogel coating, making it impossible to form a stable structure during the heating or combustion process [7,20,21,22,23].

### 2.4. Flame Retardancy of Coated RPUF

**Figure 6:**
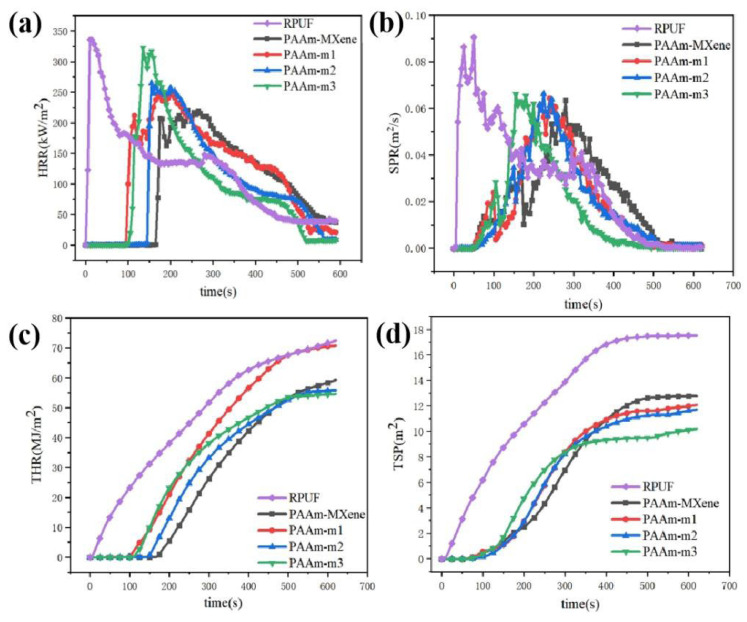
(**a**) HRR curve; (**b**) SPR curve; (**c**) THR curve; (**d**) TSP curve of pure RPUF and Coated RPUF samples.

Cone calorimetry can quantitatively evaluate the flame-retardant properties of Coated RPUF, and is often used to evaluate the combustion of materials in real fires on a laboratory scale. Among all of the data, heat release rate (HRR), peak heat release rate (pHRR), total heat release (THR), and ignition time (TTI) are important parameters for evaluating whether a material is flammable. Its test results and related data are shown in Table 1 and Figure 6.

From Figure 6a, compared with Coated RPUF, RPUF is quickly ignited (TTI is 7 s), and the TTI of Coated RPUF is greatly improved. The PAAm-MXene shows the TTI was extended to 172 s, but the ignition time of the Coated RPUF with the addition of m-MXene was shorter. The HRR values of RPUF and Coated RPUF rose rapidly and reached the peak value, but the pHRR of the Coated RPUF samples was lower than that of RPUF, while the pHRR of Coated RPUF added with m-MXene was higher than that of PAAm-MXene and increased with the addition of m-MXene. This is because m-MXene is grafted with C=C double bonds, which increases the spacing between the lamellae and makes it more dispersed in the acrylamide hydrogel coating system, which creates the thermal conductivity of nanomaterials faster. The “negative effect” of flame retardancy is more easily manifested. At the same time, the content of m-MXene affects the heat transfer rate, which explains why the pHRR of Coated RPUF with the addition of m-MXene increases with the increase in the addition amount.

From Figure 6c, the THR of Coated RPUF is lower than that of RPUF. From the THR of PAAm-m1, PAAm-m2, and PAAm-m3, with the increase in m-MXene content, the THR showed a downward trend. At the same time, the THR of PAAm-m2 is smaller under the same content of added nanomaterials (PAAm-MXene and PAAm-m2), which may be because m-MXene has better dispersibility than MXene so that the formed char layer can be better protection for internal substrates.

In addition, the smoke generation rate (SPR) and total smoke production (TSP) are important parameters for evaluating the smoke production of materials. Most casualties in fires are due to inhalation of smoke leading to poisoning or respiratory burns, so the amount of smoke production is important to evaluate the fire hazard of materials. Its related data can be shown in Figure 6.

From Figure 6b, the RPUF releases a large amount of smoke and quickly reaches the peak of the smoke generation rate when it is ignited. The smoke release of the Coated RPUF is released slowly, and the peak value of the SPR of the Coated RPUF is also lower than that of the RPUF. It is proved that the flame-retardant coating can effectively play the role of smoke suppression. Moreover, for the Coated RPUF with the addition of m-MXene, the peak of SPR arrives earlier with the increase in the m-MXene addition, which is caused by nanoparticles in the hydrogel. This result is consistent with the change of HRR, caused by “negative effects” of MXene in the coating.

From Figure 6d, the Coated RPUF with m-MXene has a significant decrease compared with the RPUF added with MXene, which indicates that the flame-retardant coating of the m-MXene layer has a better smoke suppression effect than the flame-retardant coating of MXene. Secondly, with the increase in the addition amount of m-MXene, the TSP showed a downward trend, which indicated that the addition amount of m-MXene affected the smoke suppression performance of the coating.

From the results of the cone calorimetry test, the surface modification of MXene reaches the ignition time of the flame-retardant coating earlier, and the dispersibility of MXene may be due to the surface modification of MXene, so that the heat release rate of Coated RPUF increases. However, the surface modification of MXene can reduce the THR of Coated RPUF and greatly reduce the smoke release of Coated RPUF during combustion; the more MXene that is added, the better the smoke suppression performance.

**Figure 7:**
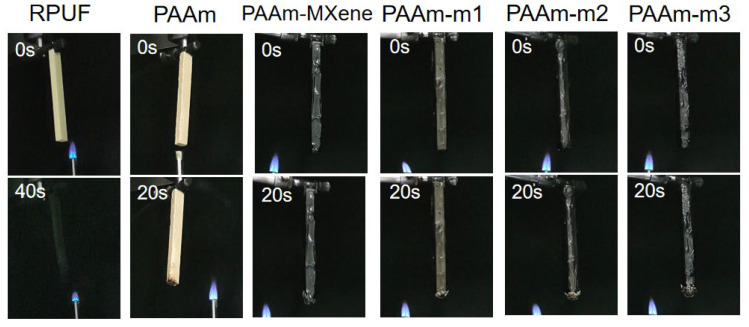
Optical photographs of pure RPUF and Coated RPUF samples before and after UL-94 testing.

In the process of vertical combustion (as shown in Figure 7), RPUF burns violently and its flame spreads rapidly throughout the material. After removing the ignition source, the flame does not self-extinguish but continues to burn until complete combustion. In comparison, the Coated RPUF was not ignited during both firings, but the PAAm Coated RPUF was partially burned and the rest retained its original shape. Compared with PAAm-m1, PAAm-m2 and PAAm-m3, PAAm-m2 has the smallest burning trace, which is consistent with the results of the cone calorimetry test and thermogravimetric test. Compared with PAAm-m2, PAAm-MXene has smaller burning traces, which may be due to the poor dispersion of MXene in PAAm-MXene hydrogel, which makes the char formation during combustion not effective on the flame contact surface which can retard the transfer of heat and play a flame-retardant effect.

**Figure 8:**
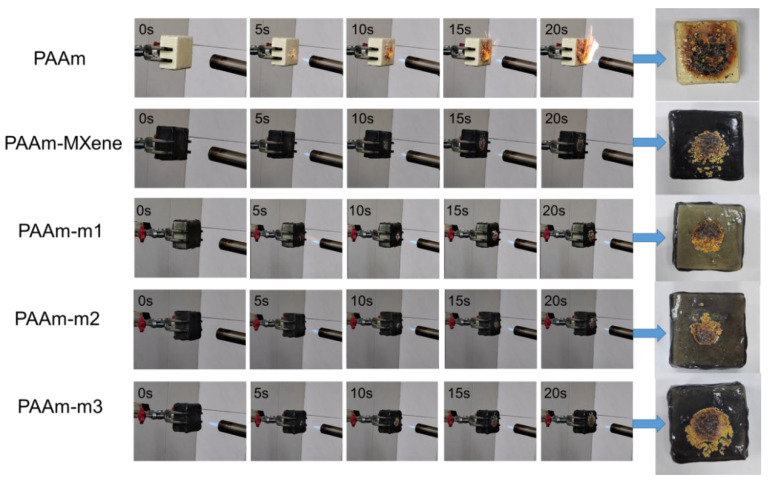
Optical photos of pure RPUF and Coated RPUF samples in the open flame combustion test.

The open flame combustion test in this experiment adopted the experiment of igniting the spray gun (Figure 8), and the final state of the coating was analyzed during the process of igniting for 20 s. All the samples were not ignited during the 20 s process of the gun ignition, but the surface of the PAAm Coated RPUF had an open flame, while the nanocomposite hydrogel Coated RPUF with nanomolecules added no open flame during the combustion process. Secondly, after burning, the flame-retardant coating of PAAm Coated RPUF was perforated and the inner matrix was leaked. At the same time, it could be found that some RPUF damage and PAAm hydrogel jointly formed a char layer. In summary, it can be observed that the nanocomposite hydrogel coating exhibits better flame-retardant properties than the PAAm hydrogel coating. From the analysis of the area of the finally formed char layer, it can be found that the area of the char layer formed by PAAm-m2 Coated RPUF is the smallest, and the area of the char layer formed by PAAm-m3 Coated RPUF is larger than that of the rest of the nanocomposite hydrogels, which is consistent with the analysis of cone calorimetry.

It can be seen from the open flame burning test that there will be bubbles in the coating during the burning process. This may be because the water in the hydrogel absorbs heat and turns into water vapor during the burning process, and it may also be burning. During the process, the hydrogel is thermally decomposed to generate gas. The generation of bubbles may cause the coating to fall off, so whether the coating has excellent mechanical properties is related to whether the coating can better adhere to the RPUF, so that the coating of Coated RPUF can protect the internal base during the combustion process. 

### 2.5. Tensile Properties of Coatings

**Figure 9:**
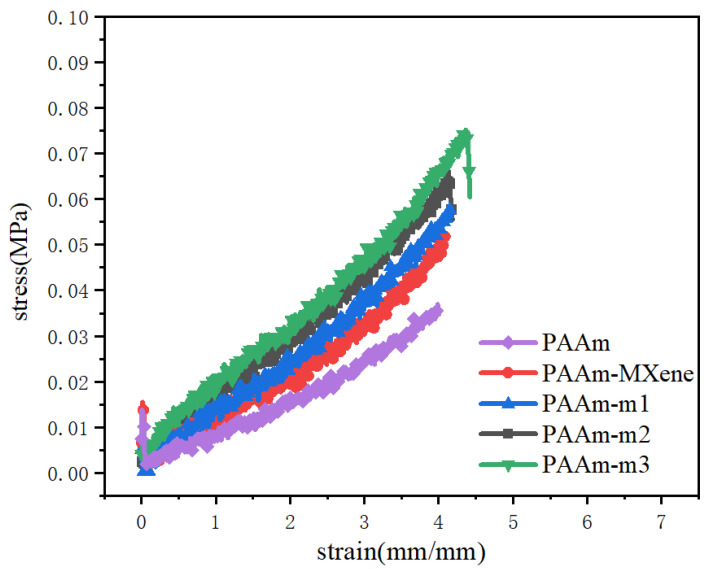
Stress-strain curve of coating tensile.

The tensile properties of the coatings were studied, and the main data are shown in Figure 9. The addition of MXene has greatly improved the tensile properties of the coating, and the elongation and fracture stress have been greatly improved, which indicates that the introduction of MXene strengthens polyacrylamide hydrogel. From the comparison of PAAm-MXene and PAAm-m2, the surface functional modification of MXene increases the tensile strength and elongation of the coating, and the overall performance is that PAAm-m2 has better tensile properties. This may be because the surface functional modification of MXene can improve the crosslinking of MXene and polyacrylamide hydrogel, and the dispersibility of modified m-MXene in polyacrylamide hydrogel reduces the agglomeration. The comparison of PAAm-m1, PAAm-m2, and PAAm-m3 shows that with the increase in the m-MXene addition, the elongation and tensile strength of the coating increase, and the tensile properties of the coating are better. The increase in the amount of m-MXene increases the crosslinking of the coating, and the carbon–carbon double bonds on the surface of m-MXene make more hydrogen bonds inside the polyacrylamide hydrogel, the tensile properties of the coating are thus better.

**Figure 10:**
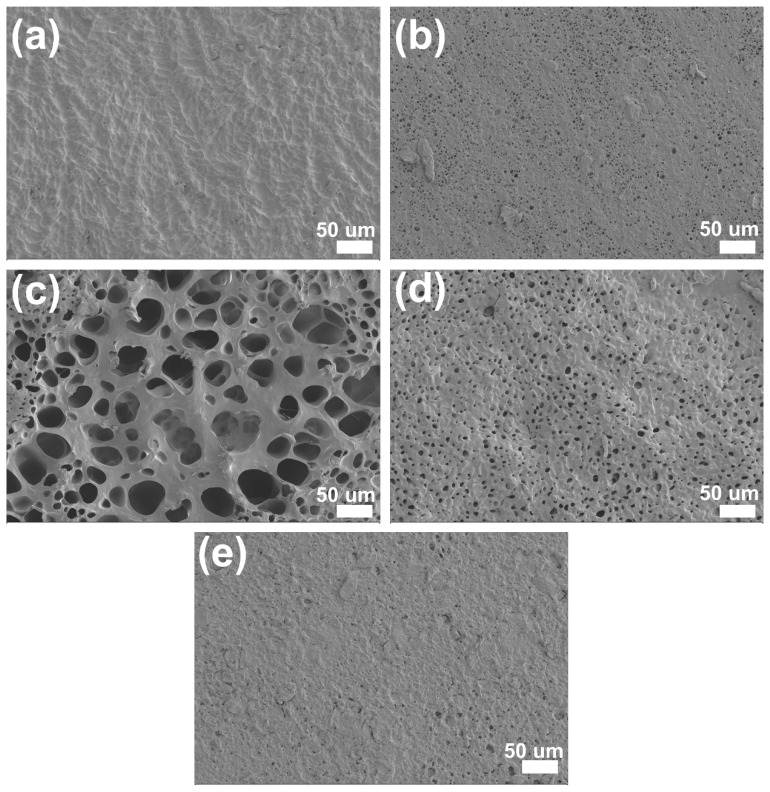
Surface SEM images of (**a**) PAAm; (**b**) PAAm-MXene; (**c**) PAAm-m1; (**d**) PAAm-m2; (**e**) PAAm-m3.

This is also consistent with the SEM image of the coating surface. The three-dimensional network porous structure can be clearly seen from Figure 10. Hydrogen bonds are formed between polar functional groups, and these pore structures provide channels for the transport of water. The formation of hydrogen bonds can greatly improve the toughness of the coating, thus further explaining the change of the stress-strain curve of the coating stretching.

## 3. Materials and Methods

### 3.1. Materials

Titanium aluminum carbide (purity 99 wt.%, 400 mesh), was provided by Laizhou Kaien Ceramic Materials Co., Ltd. (Laizhou, China). Lithium fluoride (white powder, pure) and isocyanoethyl methacrylate (colorless transparent liquid, 98 wt.%) were all provided by Shanghai McLean Biochemical Technology Co., Ltd. (Shanghai, China). Hydrochloric acid (colorless transparent liquid, 36.0~38.0 wt.%) was provided by Guangzhou Chemical Reagent Factory (Guangzhou, China). N,N-Dimethylformamide (DMF) (colorless transparent liquid, pure), was supplied by Shanghai Runjie Chemical Co., Ltd. (Shanghai, China). Anhydrous ethanol (colorless transparent liquid, pure) was supplied by Tianjin Fuyu Fine Chemical Co., Ltd. (Tianjin, China). Acrylamide was supplied by Shanghai McLean Biochemical Technology Co., Ltd. (Shanghai, China). N,N-Methylenebisacrylamide and ammonium persulfate were provided by Shanghai Aladdin Biochemical Technology Co., Ltd. (Shanghai, China). Tetramethylethylenediamine was supplied by Shanghai Yien Chemical Technology Co., Ltd. (Shanghai, China).

### 3.2. Synthesis of Functionalized MXene (m-MXene)

**Figure 11:**
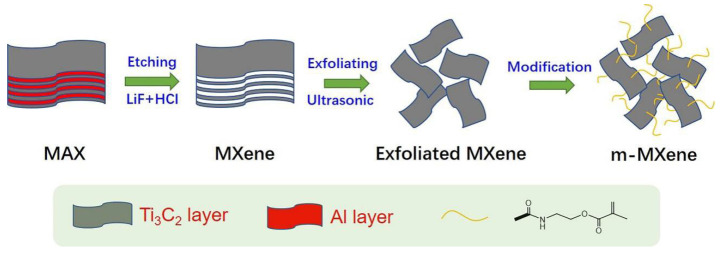
Schematic diagram of the fabrication process of functionalized MXene nanosheets.

The synthesis of m-MXene can be observed in Figure 11. A mixed solution of lithium fluoride (LiF, white powder, pure) and hydrochloric acid (colorless transparent liquid, 36.0~38.0 wt.%) was used as an etchant to etch titanium aluminum carbide. First, 4 g LiF was dissolved in 80 mL HCl (9M) to prepare an etching solution of titanium aluminum carbide by stirring. Then, 30 min later, 4g titanium aluminum carbide was slowly added to the above etching solution in 15–20 min. After 24 h of reaction under magnetic stirring, the suspension was washed with deionized water (DI) by centrifugation (3500 rpm, 5 min) for several times until the pH of the supernatant became 5. The purpose of this step was to wash off the surface of titanium carbide. Next, the titanium carbide was mixed with absolute ethanol, sonicated for 2 h, and centrifuged (10,000 rpm, 10 min) to obtain sediment. In order to exfoliate and delaminate the titanium carbide flakes to obtain MXene nanosheets, deionized water was added to the sediment, and a suspension containing MXene nanosheets was obtained after centrifugation (3500 rpm, 5 min).

The modifier used to modify the surface of MXene was isocyanoethyl methacrylate. The experimental steps were as follows: A total of 0.1 g of MXene was firstly dispersed in DMF and ultrasonicated in ice bath for 30 min to obtain a colloidal suspension. Then, 3 g of isocyanoethyl methacrylate was slowly added to the above solution under the same conditions. After 4 h of reaction, the suspension was centrifuged and the sediment was collected, and m-MXene was obtained after vacuum drying at room temperature for 24 h.

### 3.3. Preparation of Coated RPUF

MXene or m-MXene powders were firstly dispersed in deionized water, and then ultrasonicated for 30 min to obtain an aqueous dispersion. Then, AM, BIS, and APS were added to the above dispersion according to the composition formula (Table 2). N,N-methylenebisacrylamide (BIS) was used as the initiator, and ammonium persulfate (APS) was used as the cross-linking agent. In the obtained colloidal suspension, 2 mL of the solution was taken into a small tube under the condition of ultrasonic stirring in an ice bath, and 2 drops of TEMED were added and mixed thoroughly to prepare the hydrogel nanocomposite coating precursor solution.

**Figure 12:**
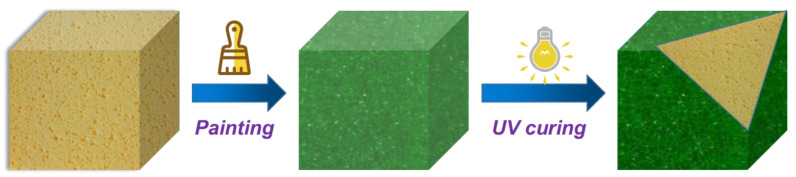
Preparation process of Coated RPUF.

The surface of the RPUF was repeatedly washed with distilled water, and then dried in an oven at 60 °C. The purpose of this step was to remove the residue on the surface of the RPUF, so that the hydrogel could better adhere to the RPUF. Next, the hydrogel coating precursor liquid was coated on the surface of RPUF and cured under UV lamp for 4 min. The above steps were repeated to coat the six sides of the RPUF with a hydrogel coating, and finally the Coated RPU was obtained. This experiment controls the thickness of the coating by the amount of solution applied and the coating times. The preparation process is shown in Figure 12.

### 3.4. Characterization

The nanoparticle composition of MXene and m-MXene was analyzed by X-ray diffraction (XRD), and their chemical structure was analyzed by Fourier transform infrared spectrometer (FTIR; model VERTEX 70; Bruker, Germany), and the experimental test wavenumber range was 400–4000 cm^−1^ with a resolution of 4 px^−1^.

The morphologies of MXene and m-MXene were observed with a scanning electron microscope (SEM; model Merlin; Zeiss, Germany) with a resolution of 0.8 nm and an acceleration voltage of 5 kV.

The morphology of the Coated RPUF was observed with a scanning electron microscope (SEM; model Merlin; Zeiss, Germany) with a resolution of 0.8 nm and an acceleration voltage of 5 kV.

Thermogravimetric analysis was performed using a thermogravimetric analyzer (model TG209; NETZCH) in a nitrogen atmosphere from 30 °C to 800 °C at a heating rate of 20 °C/min.

The UL-94 vertical burning test was conducted on CFZ-2 type instrument supplied by China Jiangning Analytical Instrument Company, the sample size was 130 × 13 × 10 mm^3^, and the test was carried out according to ASTM D 3801 standard.

The cone calorimeter test was carried out on a cone calorimeter according to ISO 5660-1 with a heat flux of 50 kW/m^2^. The sample size was 100 × 100 × 25 mm^3^.

The size of the samples in the open flame combustion test was 30 × 30 × 25 mm^3^, and the combustion test was carried out with a spray gun flame at room temperature, keeping the sample perpendicular to the flame, and the distance between the center of the sample and the flame was 5 cm.

The tensile strength was measured by a tensile testing machine, and the sample size was a dumbbell-shaped sample with a size of 50 mm × 4 mm × 2 mm, and a tensile speed of 300 mm/min.

The water retention test was carried out indoors, and the moisture change of the coating was verified by testing its weight change under actual application conditions.

## 4. Conclusions

In conclusion, a flame-retardant hydrogel coating based on organic-modified MXene was proposed and successfully fabricated. The hydrogel/MXene coating can significantly reduce the maximum weight loss rate of RPUF and improve the flame-retardant performance of RPUF. The surface functional modification of MXene enhance its dispersion, and reduces the total heat release of RPUF and the smoke release of the RPUF during combustion due to the evaporation of water, charring formation, catalytic effects and the physical barrier effect of MXene nanosheets, which protect the RPUF substrate from burning. The RPUF coating also has excellent mechanical properties. The addition of MXene improves the tensile properties of the coating by 28.7%. The combination of hydrogel and m-MXene achieves a novel flame-retardant RPUF with good smoke suppression.

## Figures and Tables

**Table 1:** The data of pure RPUF and Coated RPUF cone calorimetry test.

Samples	TTI (S)	pHRR (kW/m^2^)	THR (MJ/m^2^)
RPUF	7	335.7	72.4
PAAm-MXene	172	220.4	59.3
PAAm-m1	101	249.0	70.8
PAAm-m2	154	265.0	55.9
PAAm-m3	113	322.5	54.6

**Table 2:** Designation and formulations of the hydrogel coating.

Sample	AM (g)	BIS (mg)	APS (mg)	MXene (mg)	m-MXene (mg)	Water (mL)
PAAm	2.5	5	50	-	-	10
PAAm-MXene	2.5	5	50	10	-	10
PAAm-m1	2.5	5	50	-	5	10
PAAm-m2	2.5	5	50	-	10	10
PAAm-m3	2.5	5	50	-	15	10
